# Single Modality vs. Multimodality: What Works Best for Lung Cancer Screening?

**DOI:** 10.3390/s23125597

**Published:** 2023-06-15

**Authors:** Joana Vale Sousa, Pedro Matos, Francisco Silva, Pedro Freitas, Hélder P. Oliveira, Tania Pereira

**Affiliations:** 1 Institute for Systems and Computer Engineering, Technology and Science (INESC TEC), 4200-465 Porto, Portugal; 2 Faculty of Engineering (FEUP), University of Porto, 4200-465 Porto, Portugal; 3 Faculty of Science (FCUP), University of Porto, 4169-007 Porto, Portugal

**Keywords:** deep learning, multimodality, feature fusion, lung cancer, CT scan, clinical data

## Abstract

In a clinical context, physicians usually take into account information from more than one data modality when making decisions regarding cancer diagnosis and treatment planning. Artificial intelligence-based methods should mimic the clinical method and take into consideration different sources of data that allow a more comprehensive analysis of the patient and, as a consequence, a more accurate diagnosis. Lung cancer evaluation, in particular, can benefit from this approach since this pathology presents high mortality rates due to its late diagnosis. However, many related works make use of a single data source, namely imaging data. Therefore, this work aims to study the prediction of lung cancer when using more than one data modality. The National Lung Screening Trial dataset that contains data from different sources, specifically, computed tomography (CT) scans and clinical data, was used for the study, the development and comparison of single-modality and multimodality models, that may explore the predictive capability of these two types of data to their full potential. A ResNet18 network was trained to classify 3D CT nodule regions of interest (ROI), whereas a random forest algorithm was used to classify the clinical data, with the former achieving an area under the ROC curve (AUC) of 0.7897 and the latter 0.5241. Regarding the multimodality approaches, three strategies, based on intermediate and late fusion, were implemented to combine the information from the 3D CT nodule ROIs and the clinical data. From those, the best model—a fully connected layer that receives as input a combination of clinical data and deep imaging features, given by a ResNet18 inference model—presented an AUC of 0.8021. Lung cancer is a complex disease, characterized by a multitude of biological and physiological phenomena and influenced by multiple factors. It is thus imperative that the models are capable of responding to that need. The results obtained showed that the combination of different types may have the potential to produce more comprehensive analyses of the disease by the models.

## 1. Introduction

Lung cancer is the leading cause of cancer-related deaths, being responsible for approximately over 2 million new cases and 1.8 millions deaths in 2020 [1]. Despite the increasing risk of developing cancer related with age, tobacco consumption persists as the main contributor for all major histological types of lung cancer, accounting for about 80% of cases [2,3,4]. Nevertheless, there are other risk factors that can have a key role as well in the development of this condition, such as exposure to air pollution and second-hand smoke, occupational exposure, a diet poor in nutrients, alcohol consumption, genetic susceptibility and positive family history of lung cancer [3,4]. Given the lack of clear and distinct symptoms at early stages, when this condition begins to manifest itself in a more evident manner, by the time patients are diagnosed, lung cancer is usually in an advanced stage, and, as result, the 5-year survival rate is low, around 19%. On the contrary, if the disease is detected at earlier stages, the 5-year survival rate can increase up to 54% [5], reinforcing, for this reason, the urgent need for screening and prevention measures.

In the clinical practice, it is common for physicians to take into account the information obtained from multiple sources, namely imaging findings, clinical and demographic data, and family history, in order to give an accurate diagnosis for the patient. Through the visual inspection of medical images, such as computed tomography (CT) scans, radiologists search for evidence of lung cancer, and in case there is a suspicion of the presence of malignant nodules, patients are submitted to biopsy, an invasive procedure with associated risks. However, very often, false positives are identified, leading to unnecessary procedures in patients that are cancer-free. Furthermore, given the great amount of medical images to analyze and because physicians cannot overlook them, this task becomes time demanding, exhausting and human-error prone [6]. Artificial intelligence (AI) based methods can assist the practitioners in the correct classification of these nodules, helping to decrease the high rates of false positives and negatives, and give more accurate diagnoses for these patients. Nonetheless, the vast majority of available methods use a single modality for the task of classification, mainly imaging modalities [2], which may put constraints on the learning process of the models, as they are limited to a single type of information [6].

Motivated by the variation in the size and morphology of lung nodules, Lyu et al. [7] introduced a multi-level cross ResNet that includes three sets of parallel residual blocks, each with a specific convolutional kernel size, in order to extract features at different scales. Data from the Lung Image Database Consortium (LIDC) [8] dataset were retrieved and because they contain nodules that can fall into three malignancy categories—benign, malignant and indeterminate—the authors conducted experiments for a ternary classification and a binary classification (that only considers benign and malignant nodules). Accuracies of 0.85 and 0.92 were obtained for the former and the latter experiments. Calheiros et al. [9] presented a work that studied the importance of the perinodular area for the malignancy classification of lung nodules. Radiomic features were extracted from the perinodular and intranodular regions of the 3D CT images from the LIDC database, and different combinations of the extracted features were made. The authors tested six different machine learning methods, namely decision tree, logistic regression, random forest, k-nearest neighbor (kNN), support vector machine (SVM) and extreme gradient boosting (XGBoost), with a total of 15 models, as a result of the combination of various hyper-parameters. The overall best performance was obtained with SVM trained with the set of features pertaining to the nodule, margin sharpness and the perinodular zone, having achieved an area under the receiver operating characteristics curve (AUC) of 0.91 ± 0.031. In addition, from the feature ranking analysis of the tree-based models, the results demonstrated that 6 of the 20 top features were extracted from the perinodular region, thus highlighting its relevance for the classification task. In [10], the authors developed a 3D axial-attention network for the classification of CT lung nodules, and data were retrieved from the LIDC dataset. The model presented an AUC, accuracy, precision and sensitivity of 0.96, 0.92, 0.92 and 0.92, respectively. The authors in [11] extracted features from CT images using the convolutional neural network, histogram of oriented gradients (HOG), extended HOG and local binary pattern, and tested four different algorithms: SVM, kNN, random forest and decision trees. The LIDC dataset was, once again, used for development and evaluation, and the best performance model presented an accuracy of 0.95. Liu et al. [12] proposed an architecture denominated as Res-trans networks that combines residual and transformer blocks for the lung cancer classification of CT nodules. The method is assessed in the LIDC dataset and presents an AUC of 0.96 and an accuracy of 0.93. With the aim of studying the relationship between chronic obstructive pulmonary disease (COPD), pulmonary nodules and the risk of lung cancer, Uthoff et al. [13] explored the idea of fusing clinical features (that include the data and clinical history of the patients, the diameter of the nodules, and four pulmonary function tests) with automatically extracted features from CT images (such as measurements from the whole pulmonary parenchyma, the lobe that contained nodules, and the airways). Three approaches were implemented to study the impact of these features on the developed models: using clinical features only; using imaging features only; and combining both. Mutual information optimization (IO) and least absolute shrinkage and selection operator (LASSO) were applied for feature selection, and LASSO and an ensemble neural network (ENN) were chosen as classification models. For training and evaluation, data were collected from the COPD Genetic Epidemiology Study (COPDGene) [14], Inflammation, Health, and Lung Epidemiology Study (INHALE) [15] and National Lung Screening Trial (NLST) [16] databases, and only patients with pulmonary nodules ≥ 4 mm were selected, for a total of 327 individuals. The highest performance metric, an AUC of 0.79, was achieved with the ENN when trained with both clinical and imaging features, selected with the IO method. Motivated by the possible complementarity between the information of CT images and serum biomarkers, Jing et al. [17] developed two malignancy classification algorithms for lung cancer, one for each modality, and then studied the combination of the predictions of those two algorithms to output a final one. CT scans and serum biomarkers were collected with a total of 173 patients used. For all pulmonary nodules, the malignancy was confirmed with a biopsy. A total of 78 quantitative features were extracted from the CT segmented nodules and given to a SVM classifier. Five serum biomarkers were investigated (squamous cell carcinoma antigen (SCC); carcinoembryonic antigen (CEA), cytokeratin fragment 21-1 (CYFRA21-1); cancer antigen 15-3 (CA15-3); and carbohydrate antigen 19-19 (CA19-9) ) and also given to a SVM. As for the combination of predictions of the two algorithms, three fusion methods were studied: minimum score between the two predictions; maximum score between the two predictions; and an weighted average of the two, in which the weights assigned vary between 0.1 and 0.9. The imaging model demonstrated higher performance metrics than the biomarker model, and the maximum AUC, 0.85 ± 0.03, was obtained by combining the predictions of the two models with weight factors of 0.3 and 0.7, respectively.

As mentioned above, in a clinical setting, data from a variety of sources are considered for lung cancer diagnosis. On the other hand, a great number of current AI approaches makes use of a single data modality, with the LIDC dataset being one of the most commonly used datasets for the development of image-based models [7,9,10,11,12], as it includes labeled nodules, yet no other data modalities are provided. In more recent years, multimodal approaches applied to the biomedical field have emerged, and often deep fusion methods surpass the performance of unimodal strategies [18]. Lung cancer is a complex disease, characterized by a multitude of biological and physiological phenomena and influenced by multiple factors. Multimodality data represent the possibility of developing learning models that are capable of responding to that need. With that in mind, the goal of this work was to study and compare lung cancer classification models that are dependent on a single modality with models that translate the clinical context by integrating information from different modalities, and with that, ascertain if improvements are registered when a broader view and analysis of the patients are taken into account. Furthermore, experiments were conducted with the NLST dataset [16] since it allows the combination of those modalities and it contains more challenging cases (as seen by the results obtained in [13]) which may enable the development of a more comprehensive analysis by the learning models.

## 2. Materials and Methods

In this section, the data used and the methods implemented in this work are described. Section 2.1 gives a detailed description of the dataset used and the pre-processing steps applied, whereas Section 2.2 describes the methodologies implemented, namely the single-modality approaches in Section 2.2.1 and the multimodality approaches in Section 2.2.2.

### 2.1. Dataset

#### 2.1.1. National Lung Screening Trial

The NLST [16] was a clinical trial conducted in partnership between the Lung Screening Study group and the American College of Radiology Imaging Network, with the aim of ascertaining whether the use of low-dose helical CT for lung cancer screening in high-risk patients would reduce mortality in comparison to chest radiography. For that reason, individuals with ages between 55 and 74 and considered high-risk (current or former smokers with 30 years or more of cigarette pack smoking history) were randomly assigned to one of two possible study arms: one in which participants were scanned with chest radiography, and another in which CT was used as a screening imaging modality. Given the scope of this work, the focus was on participants who were screened with CT, and, as such, data regarding those participants were retrieved (representing a subset of the entire dataset), which included CT images, abnormalities annotations and lung cancer screening results, as well as participant data.

#### 2.1.2. CT Scans

**Figure 1:**
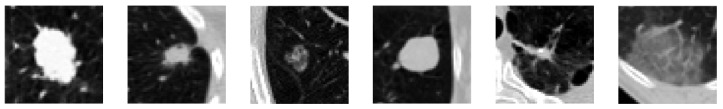
Example of bounding boxes of the CT slices of the NLST dataset. From left to right, the first three images correspond to malignant nodules, whereas the last three images correspond to benign nodules [16].

The CT scans provided were acquired with different equipment and scanning protocols, which resulted in differences in slice thickness and pixel spacing. For this reason, and to ensure homogeneity across all images, resampling was applied that set the pixel spacing to 1 mm in axes x, y and z. Afterwards, images were resized to a dimension of 128 × 128 pixels and submitted to a *min–max* normalization, with −1000 and 400 Hounsfield Units (HU) defined as lower and upper limits, to transform the original range of HU intensities to a range of [0, 1]. In the end, each scan had a dimension of 128 × 128 × s, in which *s* represents the number of slices for that scan. This dataset does not provide the segmentation masks of the identified nodules; thus, 20 × 50 × 50 bounding boxes containing the nodule in their center were manually created, with a total of 1079 3D nodule regions of interest (ROI) obtained, from which 655 were of the benign class and 424 of the malignant class. For some of the patients, more than one nodule was identified, and thus, the 1079 cases represented, in fact, a total of 1005 patients. Examples of the bounding boxes of CT slices in axial view are presented in Figure 1.

#### 2.1.3. Clinical Features

The NLST dataset also provides participant data with regards to the study in which they were enrolled; participant identifier demographics (such as age, height, weight and education); smoking habits; screening; invasive procedures and possible complications; lung cancer results; last contact; death; occupational exposure to pollutants and prevention measures; medical history; cancer history; family history of lung cancer; alcohol habits; and lung cancer progression. Given the fact that some of these features were related to lung cancer screening results and further outcomes, they were discarded in the feature selection process, in order not to introduce bias during the learning of the models, and, as a result, a total of 136 features, out of the original 324, were selected, under the following tags: demographic, smoking, work history, disease history, personal cancer history, family history, and alcohol.

#### 2.1.4. Summary

As explained above, the number of participants differs from the number of CT volumes of nodules obtained since for some patients, there was more than one nodule identified; hence, the distribution of classes benign and malignant of the CT scans and clinical data is different as presented in Table 1.

### 2.2. Methodology

**Figure 2:**
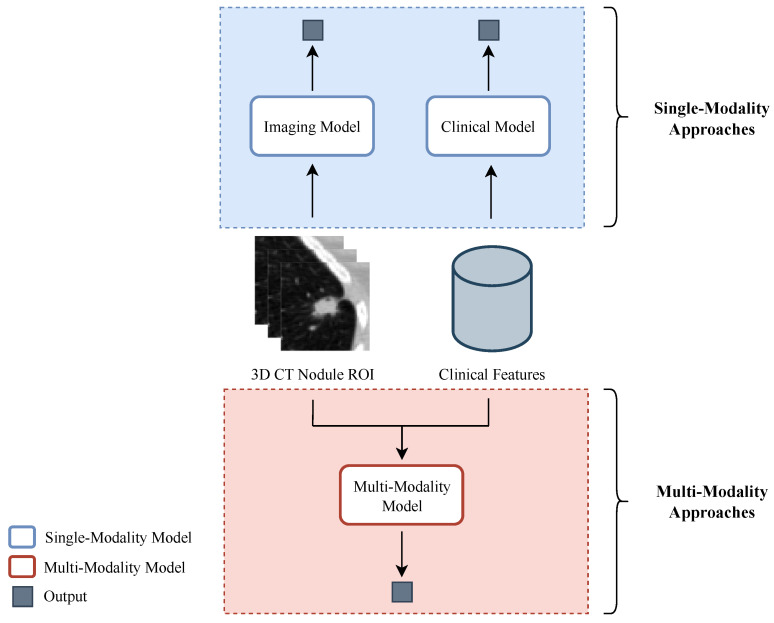
Overview of the pipeline implemented for study and comparison of the single- and multimodality strategies for lung cancer classification. Concerning the single-modality approaches, a classification model was developed for each of the data types utilized: an imaging model for the 3D CT nodule regions of interest and a clinical model for the clinical data. In the multimodality approaches, there is a fusion of the information from the two modalities.

Firstly, each data modality, the CT scans and the clinical features, was analyzed separately with the purpose of investigating its individual effect on the classification task. Afterwards, three different strategies that combine both modalities were implemented to study whether joining information from different sources is beneficial and complementary to the learning of the models. An overview of the pipeline implemented is depicted in Figure 2.

In all experiments, for the division of the data into training and evaluation, the identifiers associated with the nodules were considered, with 80% used as training data and the remaining 20% for testing. As for the clinical data, their division was made by taking into consideration the task previously assigned to the respective nodule(s), see Table 1. With the goal of identifying the best combination of hyper-parameters, 5-fold cross validation was implemented, using 80% of the data assigned for training. In this implementation, for each combination of hyper-parameters, the 80% was divided 5-fold. Four were used for training (64% of complete data), whereas the remaining was used for evaluation (16% of complete data). The process was repeated five times, and an average AUC was obtained. After all combinations were evaluated, the optimal parameters were selected as the ones that obtained the highest AUC. At last, the network was trained with the selected optimal set of parameters and using the 80% of the data assigned for training. AUC was used as a performance metric [19], and binary cross entropy (BCE) was used as the loss function.

#### 2.2.1. Single-Modality Aproaches

With respect to the imaging data, a 3D ResNet-18 architecture was chosen, given its proven efficiency in classification tasks. In the search for an optimal combination of hyper-parameters, a 5-fold cross-validation was performed. The values used for the optimizer—learning rate, batch size, dropout, and weight decay—are presented in Table 2. When employing the 5-fold cross validation, it was ensured that nodules belonging to the same patient were assigned to the same fold and that no data leakage occurred. The models were trained for 50 epochs.

As for the clinical data, the random forest algorithm was chosen since it allows the identification of the features to which more importance was given by the models. A grid search with a 5-fold cross-validation strategy was implemented, using the AUC as a scoring metric, and the parameters and respective values analyzed are presented in Table 3. After assessing the impurity-based feature ranking produced by the highest-performing model, the scope of features was narrowed down to 42. These features are as follows: demographic (age, educat, ethnic, height, marital, race, and weight); smoking (age_quit, cigar, pkyr, smokeage, smokeday, and smokeyr); work history (yrsasbe, yrsbutc, yrschem, yrscott, yrsfarm, yrsfoun, yrspain, and yrssand); disease history (ageadas, agechas, agechro, agecopd, agediab, ageemph, agehear, agehype, agepneu, agestro, diagchas, diagchro, and diagpneu); personal cancer history (ageoral and cancoral); and alcohol (acrin_drink24h, acrin_drinknum_curr, acrin_drinknum_form, acrin_drinkyrs_curr, acrin_drinkyrs_form, and lss_alcohol_num).

#### 2.2.2. Multimodality Approaches

**Figure 3:**
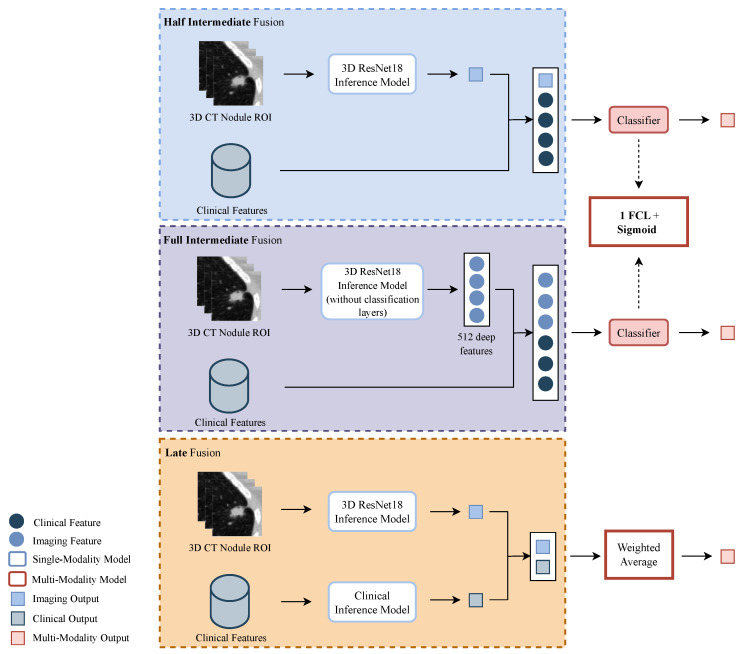
Overview of the pipeline implemented for the multi-modalities strategies. From top to bottom: half intermediate fusion (HIF), with the fusion of the imaging output and clinical features; full intermediate fusion (FIF) with the fusion of deep imaging features and clinical features; and late fusion (LF) with the fusion of the outputs given by the imaging and clinical models. For the HIF and FIF approaches, the lung cancer classification is given by a classifier constituted by one fully connected layer (FCL). In the LF approach, the classification is a weighted average of the predictions of the single-modality models.

Regarding the fusion of the two modalities, there are three main strategies that can be implemented: early fusion, in which the raw data from two or more modalities are combined and given to a single model; intermediate fusion, in which features from each modality are extracted, concatenated, and given to a single model; and late fusion, in which the final classification output is a combination of the outputs given by each modality model [20]. In order to better exploit the information inherent to each modality and because the CT volumes and the clinical features present distinct formats, the early fusion was discarded, and priority was given to the intermediate and late fusion approaches. Figure 3 depicts the pipeline implemented for the three multimodality strategies.

In relation to the intermediate fusion, two methods were studied: one denominated half-intermediate fusion (HIF), in which the malignancy probability of the volumes of the nodules, given by an inference model (the ResNet18 imaging model that achieved the highest AUC), was fused with the clinical features; and full intermediate fusion (FIF), in which 512 deep imaging features of the volumes of the nodules, given by the last layer prior to the classification layer of that same inference model of the HIF, are fused with the clinical features. In both, the concatenated features are fed to one fully connected layer (FCL), followed by a sigmoid activation layer that outputs the final probability. Furthermore, with respect to the clinical features used, two different sets were tested: one with the original 136, and another with the selected 42, as described above. A 5-fold cross validation was performed in the search for the optimal parameters. The hyper-parameters implemented are presented in Table 2. The models were trained for 200 epochs.

As for the late fusion (LF) approach, the weighted average of the outputs of the imaging model and the clinical model was computed and used to estimate the malignancy. The weight assigned to each output ranged between 0.1 and 0.9.

## 3. Results and Discussion

This section includes the results obtained for the strategies developed and further discussion.

Table 4 presents the results of the models that demonstrated the best performance for each one of the five methods studied, as well as the number of features used, in the cases in which they were necessary. From the results of the 5-fold cross-validation implementation, for the single-modality approaches, the mean AUC and standard deviation obtained for the image and clinical models were, respectively, 0.7227±0.0311 and 0.5924±0.0188. As for the intermediate fusion approaches, mean AUC and standard deviation of 0.9195±0.0029 and 0.8750±0.0129 were obtained for the half intermediate fusion and full intermediate fusion models, respectively. Table 5 presents the hyper-parameters for three of these models, namely the imaging model and both intermediate fusion models. As for the clinical model, the set of parameters that achieved the best performance was as follows: 300 estimators with a maximum depth of 7 and the maximum number of features given by log2. The weight of the classes is balanced, and gini was used to measure the quality of the splits. The hyper-parameters of the single-modality models of the LF approach were formerly described. The result presented in Table 4 corresponds to an image output weight of 0.8 and respective clinical model output weight of 0.2, which is the combination of weight factors that achieved the highest AUC.

It is possible to observe that the multimodality approaches are the ones that present the highest performance metric, which can indicate that combining information from different sources has the potential to improve the performance of the models, particularly in comparison with the clinical model. Nonetheless, these improvements are minimal when compared to the value obtained for the imaging model. Effectively, when analyzing the results obtained by the imaging model, one can see that the CT volumes containing the nodules lead to a higher capability to distinguish cancer from non-cancer diagnosis.

One possible explanation could reside in the fact that the clinical features used may not bring enough relevance to the learning, as made evident by the poor results obtained by the clinical model. These results are also in agreement with what one would expect since in a clinical context, the lung cancer diagnosis is not based solely on the characteristics of the patient, pertaining to personal information and medical history. Similarly, considering that the LF approach combines the predictions of the single-modality models and given the results of the clinical model, it was likely that it would present the lowest AUC among the three multimodality methodologies. Those insights are reflected as well in the results of the intermediate fusion approaches, for which the attention of the network is mostly on the imaging inputs, produced by the imaging inference model. On the other hand, the configuration of both intermediate fusion models is constituted by a single FCL, equivalent to the last layer of the imaging model, i.e., the classification layer, and it seems that this network was not able to fully capture the relationship between the clinical data and the features of the CT volumes, assuming its existence.

### Limitations

When analyzing the results presented in the literature, existing methods can reach performance metrics above 0.90 [7,9,10,11,12]. However, the LIDC dataset is used for the development and evaluation of their proposed methodologies. The usage of this dataset results in these excellent metrics since the data do not represent a realistic view of the clinical context (they contain mostly easier cases) and do not translate the full heterogeneity of lung cancer patterns. Moreover, the LIDC dataset provides nodule contours as a result of the annotation process made by experts, and these nodules are labeled into five malignancy categories that can be further subclassified as benign, malignant and indeterminate. The indeterminate nodules, in some approaches, are discarded, which may lead to higher performance metrics [7]. On the other hand, this study made use of a dataset, the NLST dataset, different than what the vast majority of the proposed algorithms used. The NLST dataset presents cases with more complex lung cancer patterns (that are, therefore, more challenging) and, in addition, it does not provide nodule annotations. The regions of interest of the nodules used in this study were generated in a manual process susceptible to human errors, with some degree of uncertainty regarding the malignancy level. Ultimately, all these factors had an impact on the learning models, resulting in lower performance metrics. Considering the work that uses a mutual dataset [13], the NLST dataset, another two datasets were used by the authors of [13], with a total of 327 participants, whereas this study used a total of 1005 participants from the NLST dataset only, and it is not possible to ascertain if the same patients were used. Moreover, in this work, regions of interest of the nodules were manually generated, which adds another layer of divergence. As such, a comparison between the two works would not be fully equitable.

Additionally, the predictive capability of the clinical features seems to be very limited, which is corroborated by the clinical practice, in which physicians use these data in an initial phase of screening in order to discern patients that may have lung cancer. Afterwards, an initial diagnosis of this pathology is given to those patients through the visual assessment of medical images and subsequently confirmed with biopsy.

## 4. Conclusions

This work aimed at investigating the combination of more than one type of information for predicting lung cancer, specifically, extracted from CT nodules and clinical data. The study of each modality and the results obtained showed the utmost importance of the imaging data, essential for lung cancer diagnosis. The clinical features used, on the contrary, demonstrated poor predictive capability when used alone, which is understandable, as they are used as complementary information in the clinical context, serving as primary suspicion in the screening stage. The results obtained from the multimodality approaches showed the potential of fusing different data modalities. The future investigation could branch out from the described work, with the possibility of combining different strategies and architectures, such as implementing deep learning approaches for the extraction of features from the clinical data, with the goal of exploiting to its maximum potential the relationship shared between two distinct modalities.

## Figures and Tables

**Table 1:** Class distribution for imaging and clinical modalities.

Data Modality	Class		Task
	# Benign	# Malignant	
CT Scans	522	339	Train
	133	85	Test
Clinical Features	463	337	Train
	121	84	Test

**Table 2:** Hyper-parameters used for the development of the imaging and intermediate fusion models.

Hyper-Parameter	Value
Optimizer	Adam, SGD
Learning rate	0.01, 0.001, 0.0001
Weight Decay	0.01, 0.001, 0.0001
Batch size	16, 32, 64
Dropout	0.3, 0.4, 0.5, 0.6

**Table 3:** Hyper-parameters used for the development of clinical models.

Hyper-Parameter	Value
# Estimators	200, 300, 400, 500, 600
Criterion	gini, entropy
Max features	sqrt, log2
Maximum depth	3–9
Class weight	None, balanced

**Table 4:** Results obtained for the five methods implemented. The highest performance metric, highlighted in bold, is obtained for the Full Intermediate Fusion approach.

Approach		# Clinical Features	AUC
**Single-Modality**	Image Model	-	0.7897
	Clinical Model	136	0.5241
	HIF	42	0.7934
**Multimodality**	FIF	42	**0.8021**
	LF	136	0.7911

**Table 5:** Hyper-parameters of models with the highest performance metric for the image-only and intermediate fusion approaches.

Approach		Optimizer	Learning	Weight	Batch	Dropout
			Rate	Decay	Size	
**Single Modality**	Image Model	SGD	0.0001	0.001	32	0.4
**Multimodality**	HIF	Adam	0.01	0	16	0.4
	FIF	Adam	0.0001	0	64	0.5

## Data Availability

The data was obtained from the dataset National Lung Screening Trial [16].
